# Chitosan and Chitooligosaccharide: The Promising Non-Plant-Derived Prebiotics with Multiple Biological Activities

**DOI:** 10.3390/ijms23126761

**Published:** 2022-06-17

**Authors:** Zhiwei Guan, Qiang Feng

**Affiliations:** 1 Shandong Provincial Key Laboratory of Oral Tissue Regeneration, Shandong Engineering Laboratory for Dental Materials and Oral Tissue Regeneration, Department of Human Microbiome, School of Stomatology, Shandong University, Jinan 250012, China; guanzhiwei1109@163.com; 2 School of Life Science, Qilu Normal University, Jinan 250200, China; 3 State Key Laboratory of Microbial Technology, Shandong University, Qingdao 266347, China

**Keywords:** chitosan, chitooligosaccharide, prebiotic, antioxidant activity, anti-inflammatory activity, antimicrobial activity

## Abstract

Biodegradable chitin is the second-most abundant natural polysaccharide, widely existing in the exoskeletons of crabs, shrimps, insects, and the cell walls of fungi. Chitosan and chitooligosaccharide (COS, also named chitosan oligosaccharide) are the two most important deacetylated derivatives of chitin. Compared with chitin, chitosan and COS not only have more satisfactory physicochemical properties but also exhibit additional biological activities, which cause them to be widely applied in the fields of food, medicine, and agriculture. Additionally, due to their significant ability to improve gut microbiota, chitosan and COS are deemed prospective prebiotics. Here, we introduced the production, physicochemical properties, applications, and pharmacokinetic characteristics of chitosan and COS. Furthermore, we summarized the latest research on their antioxidant, anti-inflammatory, and antimicrobial activities. Research progress on the prebiotic functions of chitosan and COS is particularly reviewed. We creatively analyzed and discussed the mechanisms and correlations underlying these activities of chitosan and COS and their physicochemical properties. Our work enriched people’s understanding of these non-plant-derived prebiotics. Based on this review, the future directions of research on chitosan and COS are explored. Collectively, optimizing the production technology of chitin derivatives and enriching understanding of their biological functions will shed more light on their capability to improve human health.

## 1. Introduction

Currently, the potential biosafety and environmental threats in the application of synthetic pharmaceuticals and antibiotics are becoming increasingly serious. Seeking and exploiting active and biodegradable molecules in nature with better biosafety and biocompatibility are receiving more and more attention.

Chitin was first isolated from a mushroom by French chemist Henri Braconnot in 1811 [1]. It is the second-most abundant natural long-chain polymer, after only cellulose. Chitin is composed of *N*-acetyl-glucosamine monomers linked by a β-1,4-glycosidic bond, with the degree of polymerization (DP) ranging from about 1000 to 3000. This polymer widely exists in the exoskeleton of crabs, shrimps, and insects, as well as in the cell walls of fungi, where it forms a highly ordered crystal structure and plays a role in supporting and protecting cells [2]. Chitin with a high molecular weight (MV) and a degree of acetylation (DA) over 90% is highly hydrophobic and hardly dissolves in water and most solvents [3]. Biodegradable and environmentally friendly chitin has good thermal stability and chemical tolerance. Therefore, it can be used in many light industrial applications for different purposes [4,5]. For example, chitin can be applied as a thickener and stabilizer in the process of food or medicine production [6]. It is also frequently used as flocculant in sewage treatment and as biomaterial for tissue repairing and drug delivery [7]. However, chitin’s water insolubility and potential side effects, such as inducing eosinophilic allergic inflammation in the lungs, limit the application of chitin in life systems [8]. In order to solve these problems, chitin can be further modified into its derivatives, which have better physicochemical and physiological properties, especially solubility and biocompatibility. Chitosan and COS are the two most important chitin derivatives.

Here, we outlined the production, physicochemical properties, and pharmacokinetic characteristics of chitosan and COS. Based on reviewing the relevant research literature, especially the papers published in the past five years, we summarized the biological activities of chitosan and COS. We particularly focused on and elaborated their prebiotic effects, which have attracted much attention recently. We hope that this review can help the readers learn about the two chitin derivatives and their influence on human health. We also provide some advice for future research in related fields.

## 2. Production, Physicochemical Properties, and Applications of Chitosan and COS

Chitosan is the deacetylated product of chitin. The chitin existing in the exoskeletons of marine crustaceans (e.g., shrimp, crab, squid, and lobster), where it is closely combined with other components, such as protein, minerals, lipids, and pigments, is the traditional source for chitosan production. In order to extract pure chitosan from crustacean shell waste, severe chemical treatments are required, including removing impurities such as protein and calcium salt. Then, deacetylation is performed with concentrated aqueous alkali (e.g., 10–15 M NaOH or KOH) at a high temperature of 40 to 100 °C. However, chitosan obtained via the above treating methods suffers from such physicochemical defects as protein contamination and inconsistent levels of deacetylation and MW. Furthermore, environmental pollution, seasonal limitation of raw material supply, and high production cost are also inconvenient problems [9]. Recently, microwave irradiation combined with chemical and enzymatic methods has been used to prepare chitosan, which effectively shortens the time of chitin deacetylation [10]. Fungal mycelium is another abundant, steady, and economical source of chitin. Compared with the traditional crustacean resource, fungal mycelium has no allergenic marine crustacean protein and lower inorganic content, for which demineralization is not required when processing. Meanwhile, the extraction of chitosan from the fungi-derived chitin can be achieved via treatments using acids and alkali with very low concentrations, which has better environmental safety and is considered a feasible green method in laboratories [11]. Additionally, the preparations of chitosan by ionic liquid and via microbial fermentation or the proteolytic enzymes produced by bacterial communities are also promising and eco-friendly extraction processes [12]. When the degree of deacetylation (DD) reaches 50%, chitosan obtains water solubility and viscosity at acidic pH due to the protonation of amino groups in the polymer molecule [2]. Chitosan dissolves more easily as its DD increases. Commercial chitosan has a DD of up to 90% [13]. Based on the application requirements, chitosan can be processed into different conformations, including fiber, film, sponge, beads, gel, solution, and nanoparticles [14,15]. Compared with chitin, chitosan is not only less toxic and less allergenic but also has better adsorbability, permeability, and abilities of film-forming and moisturizing. These unique structural and physicochemical properties endow chitosan with wider application prospects in the fields of food, medicine, cosmetics, agriculture, and so on [16]. Due to the abilities of its hydroxyl and amino groups to adsorb or chelate such pollutants as dyes, metal ions, and organic compounds under different pH conditions, chitosan is usually utilized in wastewater treatment [17,18]. Chitosan is also recognized as a food additive. It can be used as an inhibitor of browning and as a clarifying agent in juices [19], as well as as a preservative and antioxidant in processed meat [20]. In plant protection, chitosan-based materials are prospective drugs against phytopathogenic fungi owing to their antifungal and biodegradability characteristics [21]. In the field of medicine, chitosan can be used as a lipid-lowering drug. First, soluble chitosan is dissolved in gastric acid and then reaches the small intestine, where it combines with cholic acid to form an insoluble complex. Next, this complex is excreted from the body through feces so as to reduce the absorption of cholic acid and lower the serum level of cholesterol [22]. Chitosan also can promote wound healing and be used as a carrier to assist the delivery and transport of macromolecular drugs, vaccines, and nucleic acids [23,24,25,26]. In recent years, chitosan, as a new biomaterial particularly in stomatology, has been applied in oral surgery, restorative dentistry, and improving periodontal disease due to its fine antimicrobial and drug-delivering properties [27]. For example, chitosan can be used in dental cements to reduce the formation of bacterial biofilm or be added to toothpastes as a demineralizer [27]. Additionally, it also shows good potential in inducing periodontal, bone, cartilage, vascular system, neural network, and skin tissue regeneration [28,29]. As the electrospun material for bioink, chitosan has been utilized to produce 3D-printed scaffolds applied in tissue engineering and drug delivery. In contrast to conventional 3D scaffolds such as gels and fibrous matrices, the chitosan-based material can recapitulate native tissues with high exactness due to its better mechanical stability, cellular adhesion, and biocompatibility [30]. Although superior to chitin, some physicochemical flaws of chitosan (e.g., low water solubility, high viscosity, slow gelation rate, and weak mechanical strength) limit its utilization. To compensate for this, the plentiful hydroxyl and amino groups on the molecule of chitosan can be modified to a certain extent via chemical methods, including acylation, carboxylation, alkylation, quaternization, and graft copolymerization, as well as physical and enzymatic methods [30,31,32,33,34,35]. These reasonable modifications of chitosan can improve such physicochemical and biological properties as solubility, thermal stability, rheology, antioxidant effect, biocompatibility, and antimicrobial effect in order to broaden its applications in various fields. In addition, as the MV of chitosan reduces, the number of hydrogen bonds in the polymer chain and between chains decreases, which makes the molecules loose and easier to dissolve [36]. Therefore, hydrolyzing the glycosidic bonds between glucosamine units in chitosan to lower the MV is a reasonable strategy for improving its solubility and application performance. Perhaps this is the reason why COS has attracted extensive attention of many researchers.

**Figure 1:**
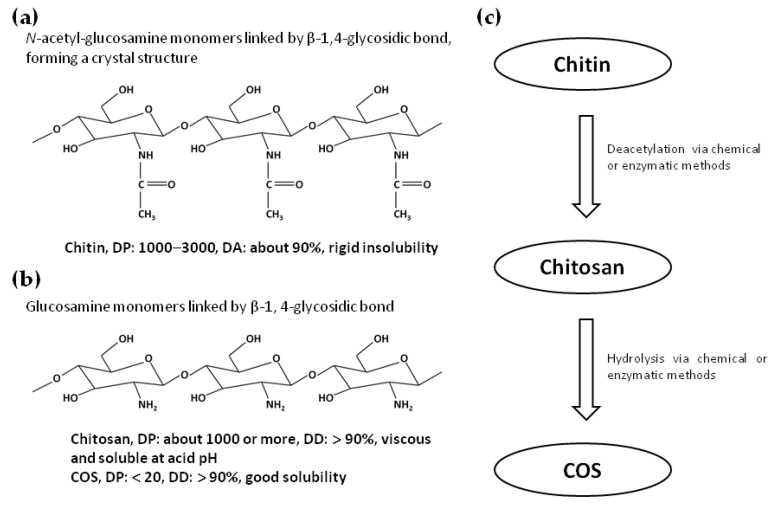
Schematic diagrams of the structures of chitin, chitosan, and COS and the process for producing chitosan and COS from chitin. (**a**) Schematic diagram of the structure of chitin. DP: degree of polymerization, DA: degree of acetylation. (**b**) Schematic diagram of the structures of chitosan and COS. DD: degree of deacetylation. (**c**) Schematic diagram of the process for producing chitosan and COS from chitin.

COS is the hydrolysis product of chitosan, which is usually defined as those polymers/oligomers with a degree of DD of more than 90%, a DP of fewer than 20, and an average Mw of less than 3.9 kDa [8]. Hydrolysis of chitosan can be carried out by chemical or enzymatic methods, among which chemical hydrolysis by acid or oxidizer is often applied in industrial production of COS [37]. In spite of the lower cost of production, chemical hydrolysis is frequently limited by the lack of an effective technique for the large-scale production of COSs with specific MVs. Additionally, the potential risks in environmental pollution and formation of toxic byproducts are also shortcomings, causing doubts and criticism [38]. As an eco-friendly technology, the enzymatic method applied in the hydrolysis of chitosan makes up for these shortcomings derived from chemical hydrolysis. This method can be manipulated under moderate conditions to produce safe and bioactive COSs. Many enzymes have been utilized in the hydrolysis of chitosan, of which chitinase and chitosanase have a high substrate specificity to chitosan. Several nonspecific enzymes such as protease, lipase, cellulase, hemicellulase, lysozyme, pectinase, and α-amylase can also be applied this process [39]. Meanwhile, the development and application of immobilized enzyme technology reduce the production cost and achieve the continuous production of COS [40]. The schematic diagram of the process for producing chitosan and COS from chitin is shown in Figure 1. COS has the excellent characteristics of chitosan. Furthermore, many studies demonstrated that COS showed different physiological functions, including antioxidant, anti-inflammatory, metabolic regulatory, antitumor, and antimicrobial activities. COS has been widely applied in biomedical, pharmaceutical, nutraceutical, and cosmeceutical production fields [38]. It is noteworthy that COS has greater solubility at neutral pH and lower viscosity than chitosan because of the low DP and MV of COS. Moreover, COS permeates through intestinal epithelial barriers more easily and exhibited better cell transduction activity than chitosan [38]. These valuable properties, including better solubility and absorbability, make COS more advantageous for certain specific clinical treatments, such as antioxidation, anti-inflammation, immunomodulation, and antitumor, than chitosan [41].

## 3. Pharmacokinetic Characteristics of Chitosan and COS

**Figure 2:**
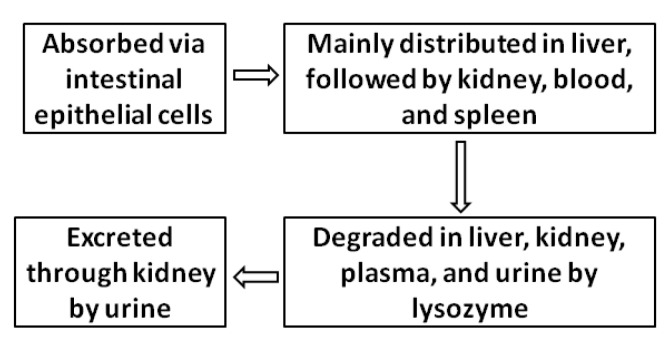
Schematic diagram of the pharmacokinetic process of chitosan and COS.

Because of the lack of digestive enzymes (e.g., chitinase, chitosanase, cellulase, hemicellulase, and pectinase) which can hydrolyze β-1,4-glycosidic bonds in human digestive fluid, chitosan and COS can hardly be degraded in the small intestine. However, based on good biocompatibility, chitosan and COS can be absorbed by the gut and further metabolized. Understanding the pharmacokinetic characteristics of chitosan and COS is of great significance for safe and efficient medical applications. The absorption and metabolism of chitosan and COS have so far been analyzed and elucidated by fluorescein isothiocyanate (FITC) labeling technology. Chea et al. investigated the impact of molecular weight on the absorption of water-soluble chitosan by both in vitro and in vivo experiments with Caco-2 cells and male Sprague Dawley (SD) rats, respectively. The results revealed that soluble chitosan and COS can be absorbed via intestinal epithelial cells, and the absorption was significantly influenced by MW, with the absorption decreasing as the MW increased. Compared with the poorly absorbed chitosan with an MV of 230 kDa, the absorption of COS with an MV of 3.8 kDa in vitro and in vivo increased by more than 23 times and 25 times, respectively [42]. Zeng et al. examined the distribution of four chitosan samples with different MWs and DDs in mice after oral administration. The results indicated that the absorbed chitosan was distributed in all tested organs, such as the liver, the kidneys, blood, the spleen, and so on. The concentration of chitosan in blood was much less, while the concentration in the liver was much higher [43]. As for the in vivo metabolism, the analyses were carried out in mice and rats with intraperitoneal administration. The results demonstrated that chitosan and COS are primarily degraded in the liver, the kidneys, plasma, and urine by lysozyme [44]. Hepatic enzymes play a dominant role in the process of degrading and metabolizing chitosan and COS. The final degradation products of chitosan and COS were primarily excreted from the body through the kidney in urine at low MWs [45]. The pharmacokinetic process of chitosan and COS is illustrated in Figure 2.

## 4. Biological Activities of Chitosan and COS

The most important chitin derivatives, chitosan and COS, not only have agreeable biodegradability, biocompatibility, biosafety, and physicochemical properties but also exhibit excellent biological activities, which provide a premise for their wide applications in the fields of medicine and industry. In this section, we focused on concerned studies, especially in recent years, and summarized the biological effects of chitosan and COS, including antioxidant, anti-inflammatory, and antimicrobial activities.

### 4.1. Antioxidant Activity

The aging of animals is closely related to the oxidative damage caused by free radicals. Many studies have confirmed that excessive free radicals in animals that are not cleared in time are an important factor leading to aging and many noncommunicable diseases (e.g., diabetes) [46]. Although free radicals in the body were found to have certain health benefits, such as immunity and signal transduction, too many free radicals will give rise to the denaturation of biological macromolecules, including protein and nucleic acid, which aggravates cell destruction and apoptosis in some pathological states [47]. The body’s ability to scavenge free radicals is limited and weakens with age. Taking exogenous free radical scavengers is an effective method to delay aging and prevent diseases related to it. Therefore, finding edible or pharmaceutic free radical scavengers beneficial to the human body is an urgent concern. Biologically safe chitin derivatives, such as chitosan and COS, are excellent natural antioxidants.

In recent years, more and more efforts have been focused on research on the antioxidant activity of chitosan and COS. For example, the antioxidant activity of chitosan in salmon was investigated by 2-thiobarbituric acid-reactive substances (TBARS) and 2, 2-diphenyl-1-picrylhydrazyl (DPPH) scavenging assays. The results revealed that the addition of chitosan to salmon reduced lipid oxidation for 15 days of storage, and chitosan exhibited comparable antioxidant activity to butylated hydroxytoluene (BHT) but is safer than the latter [48]. Brol et al. reported that chitosan reduced lipid peroxidation in the gills and hepatopancreas of cultivated shrimp subjected to salinity stress by increasing glutathione-S-transferase (GST) activities and glutathione (GSH) levels, which showed antioxidant and protective effects [49]. The results of in vitro experiments by Mi et al. showed that a chitosan derivative modified with Schiff-base-bearing benzenoid/heterocyclic moieties exhibited significantly increased antioxidant activity, with a DPPH scavenging effect which was close to that of ascorbic acid [50]. As for COS, it was found to alleviate the decrease of GSH and catalase (CAT) activities as well as the increase in malondialdehyde (MDA) levels in the organs of mice with lipopolysaccharide (LPS)-induced sepsis [51]. Qu and Han revealed that feeding with a high fat diet (HFD) containing 0.5% COS resulted in a significant increase in the activities of superoxide dismutase, CAT, and glutathione peroxidase in the stomach, liver, and serum of mice as compared with the control group, meaning that COS can exert an antioxidant effect by restoring the activities of antioxidant enzymes [52]. Likewise, the study in C57BL/6 mice indicated that COS ameliorated hepatic oxidative stress by activating the nuclear factor E2-related factor 2 (Nrf2) pathway and upregulating gene expressions of antioxidant enzymes, including GSH peroxidase (GSH-Px), superoxide dismutase (SOD), NADPH quinone oxidoreductase 1 (NQO1), heme oxygenase-1 (HO-1), and glutathione S-transferase alpha 1 (GSTA1) [53]. The results from Liu et al. showed that supplementation of COS increased superoxide dismutase activity and decreased lipid peroxidation products, which contributed to alleviating abnormal glucose metabolism in the HFD/streptozotocin-induced diabetic rat livers [54].

Although the exact mechanism of antioxidant activity is unclear, this is probably due to the reaction of energetic free radicals with amino and hydroxyl groups on the pyranose ring of chitosan and COS, which boosts the formation of stable complex molecules. Furthermore, COS has the ability to induce the activities of antioxidant enzymes when confronted with oxidative stress. In addition, Laokuldilok et al. prepared COSs with different DPs with papain and found that, compared with COSs with DPs of 14 and 41, COS with a DP of 5 had the highest antioxidant activities of all of the assays used, including the lowest 50% effective concentration (EC50) for DPPH radical scavenging activity, the highest reducing power, and the highest metal-chelating activity [55]. This suggests that the antioxidant activity of chitosan and COS depends to a certain extent on the DP or MW.

### 4.2. Anti-Inflammatory Activity

It is well known that immunity refers to the capability of the immune system to distinguish alien and non-alien substances and eliminate antigenic foreign objects through immune responses so as to maintain body homeostasis. Inflammation is a kind of severe immune response, which is the body’s physiological protection from such deleterious stimuli as pathogens and products of tissue injury. Inflammatory responses usually include the production of proinflammatory cytokines (e.g., IL-1β, TNF-α, and IFN-γ) and free radicals and the recruitment and activation of immune cells [56]. In normal physiological states, these inflammatory processes are conductive to the elimination of pathogens and foreign objects, which helps tissue repair and regeneration [57]. However, the inflammatory responses become hyperactive in certain abnormal states. Excessive production of inflammatory factors results in metabolic disorders (e.g., atherosclerosis), tissue damage (e.g., rheumatoid arthritis), and even tumor formation [58]. Chitin derivatives not only exhibit good immune activity, enhancing both innate and adaptive immune systems, but also have excellent anti-inflammatory activity [59,60].

Many animal studies supported the anti-inflammatory effect of chitin derivatives, especially COS. In LPS-challenged mice, intraperitoneal administration of COS not only combated oxidative damage but also exerted significant anti-inflammatory activity by reducing neutrophil infiltration in organs and TNF-α and IL-1β in serum [51]. A similar effect was observed in dextran sodium sulfate (DSS)-induced inflammatory bowel disease (IBD) mice model, in which oral administration of COS at a dosage of 10–20 mg/kg/day improved IBD by suppressing the nuclear factor kappa B (NF-κB) signaling pathway and decreasing the levels of TNF-α and IL-6 in colonic tissues [61]. Recently, in another DSS-induced mice model, treatment with chitosan and COS relieved the inflammation caused by ulcerative colitis (UC). The anti-inflammatory effect is characterized by the increased level of anti-inflammatory IL-10, the reduced levels of proinflammatory factors, including IL-1β, IL-6, TNF-α, myeloperoxidase, and inducible nitric oxide synthase (iNOS), as well as the inhibition of the TRL-4/NF-κB/mitogen-activated protein kinase (MAPK) signaling pathway [62]. Additionally, the administration of COS by oral gavage can reduce proinflammatory cytokines, neutrophil infiltration, and macrophage polarization in the liver of HFD-fed mice by the activation of the adenosine-monophosphate-activated protein kinase (AMPK) signaling pathway [53]. Mohyuddin et al. confirmed that oral administration of COS at a dosage of 300–600 mg/kg/day for 7 days relieved the transcriptional downregulation of tight junction proteins Claudin-2 and Occludin in colonic tissue under heat stress in mice. Further analysis displayed that this administration suppressed the production of HSP70, TLR4, p65, TNF-α, and IL-10, indicating an anti-inflammatory ability of COS to tolerate hot environmental conditions [63]. In summary, the anti-inflammatory activity of chitosan and COS is closely related to the regulation of specific signaling pathways mainly including inhibiting NF-κB and MAPK pathways and activating the AMPK pathway.

Over the past decade, the mechanisms underlying the anti-inflammatory activity of COS were explored and enriched especially by in vitro studies. Firstly, the study on the mouse RAW 264.7 macrophage cell line demonstrated that COS can block the binding of LPS to TLR4 through competitive binding with TLR4 in order to attenuate the downstream NF-κB and MAPK signaling pathways [64]. This mechanism was also confirmed by the study with intestinal porcine epithelial cells [65]. Secondly, COS can inhibit the nuclear translocation of NF-κB, which attenuates corresponding inflammatory responses. This inhibition can be achieved by suppressing C-Jun-N-terminalkinase (JNK1/2), preventing degradation of inhibitory kappa B (IκB), or reducing OGT (O-GlcNAc transferase)-dependent O-GlcNAcylation of NF-κB [66,67]. Thirdly, in LPS-stimulated RAW 264.7 cells, COS was found to alleviate the inflammatory responses through NF-E2-related factor 2 (Nrf2)/MAPK-mediated induction of heme oxygenase-1 (HO-1) [68]. Additionally, the studies in both LPS-stimulated RAW 264.7 cells and the colonic tissues of DSS-induced colitis mice indicated that COS can upregulate the level of peroxisome proliferator-activated receptor gamma (PPARγ). PPARγ directly combines to NF-κB p65, resulting in its ubiquitination and degradation or the increased expression of Sirtuin 1 (SIRT1). SIRT1 is a NAD^+^-dependent histone deacetylase, which inhibits the activity of NF-κB by deacetylaing Lys310 in NF-κB p65 [69]. Generally, COS can exert an anti-inflammatory effect via the PPARγ/SIRT1-mediated interference with the NF-κB pathway.

Due to the anti-inflammatory activity of Chitosan and COS, they exhibit significant regulatory effect on the glucolipid metabolism. The studies by Bai et al. suggested that in HFD-fed C57BL/6J mice, COS can improve glucolipid metabolism disorder in the liver by inhibiting the transcriptional expression of proinflammatory cytokines, including IL-6, monocyte chemoattractant protein-1 (MCP-1), and TNF-α, as well as upregulating the expression of PPARγ [70]. The effect of ameliorating glucolipid metabolism was also found in a diabetic rat model fed with a diet containing 5% chitosan. The mechanism is related to decreasing plasma TNF-α, insulin resistance, and the activities of alanine aminotransferase (ALT) and adipose tissue lipoprotein lipase by oral administration with chitosan [71]. One of the causes of insulin resistance is attributed to chronic tissue inflammation [72]. Kwon et al. reported that the administration of COS with an MV less than 1.0 kDa exerted antidiabetic effects by significantly reducing hyperglycemia in both animals and humans [73,74,75,76]. Recently, the same team evaluated the antiobesity effect of COS on lipid accumulation and adipogenic gene expression with 3T3-L1 adipocytes and an SD rat model, respectively. The findings suggested that COS may prevent diet-induced weight gain and that the antiobesity effect is mediated partially via inhibiting adipogenesis and increasing adiponectin levels [77]. Obesity, the most common chronic metabolic disease, has been proved to induce low-grade inflammation [78]. The studies above supported the feasibility that COS with anti-inflammatory activity can be applied as a nutraceutical against metabolic diseases such as diabetes and obesity. Meanwhile, COS was found to exhibit an antitumor effect which is largely involved with its anti-inflammatory activity. Muanprasat et al. revealed that oral administration of COS prevented the development of aberrant crypt foci in a mouse model of colitis-associated colorectal cancer (CRC) via a mechanism involving AMPK-activation-induced β-catenin suppression and caspase-3 activation. They further confirmed that COS-induced AMPK activation was probably due to calcium-sensing receptor (CaSR)-phospholipase C (PLC)-IP3-receptor-channel-mediated calcium release from the endoplasmic reticulum (ER) by in vitro experiment with T84 cells [79]. Furthermore, AMPK activation by COS suppressed the NF-κB-mediated inflammatory responses and the mammalian targets of rapamycin (mTOR) signaling [80], the latter of which play important roles in tumor cell proliferation [81]. The anti-inflammatory activity of COS makes it a potential biomaterial which can be applied in the prevention and treatment of CRC and other tumor diseases.

### 4.3. Antimicrobial Activity

Allan and Hadwiger first reported that chitosan has antifungal activity in 1979, which attracted extensive attention from researchers [82]. So far, many studies have confirmed that chitosan and COS have a broad-spectrum antimicrobial activity on bacteria, fungi, and viruses [41], but are almost nontoxic to healthy mammalians [83,84,85].

It was demonstrated that chitosan and COS showed antibacterial effects on many clinically important Gram-positive and Gram-negative bacteria, especially some pathogenic bacteria, including *Escherichia coli*, *Vibrio parahaemolyticus*, *Salmonella typhimurium*, *Pseudomonas aeruginosa*, *Mycobacterium luteum*, *Streptococcus mutans*, *Streptococcus faecalis*, *Streptococcus epidermidis*, *Staphylococcus aureus*, *Listeria monocytogenes*, and *Yersinia enterocolitica* [86,87,88]. There are several mechanisms underlying the antimicrobial activity of chitosan and COS. Firstly, they suppress bacteria via combining with the components with negative charges on the bacterial surface (e.g., peptidoglycans in the cell wall of Gram-positive bacteria or O-antigens of lipopolysaccharide in the outer membrane of Gram-negative bacteria) via their protonated amino group with positive charges at acidic pH. This electrostatic combination of chitosan and COS with bacteria prevents transmembrane transport and also results in damage and leakage of the cell surface [83]. Additionally, the free amino groups of chitosan and COS have a strong ability to chelate the metal ions on the cell surface of bacteria to form complexes, thus preventing the growth of the bacteria by cutting off the supply of minerals [89]. At the same time, this metal-chelating property can obstruct the formation of insoluble calcium phosphate so as to promote the absorption of calcium ions in the small intestine [90]. Moreover, because of low molecular weight, COS and chitosan with low DP can enter the bacterial cell and penetrate the nucleoid. The following binding with DNA interferes with its replication and expression and then destroys the intracellular metabolism of the bacteria [83,89].

These above mechanisms also explain the antifungal activity of chitosan and COS. An organometallic nanoparticle of chitosan conjugated with iron oxide exhibited significant dose-dependent antifungal activity against the phytopathogen *Rhizopus oryzae* [21]. Chitosan was also proved to suppress other phytopathogenic fungi, including *Botrytis cinerea*, *Phytophthora infestans*, *Alternaria solani*, *Fusarium oxysporum*, *Aspergillus flavus*, and *Penicillium* spp. [91,92]. Likewise, the antifungal activity of COS was analyzed by detecting the minimum inhibitory concentration (MIC). The results showed that COS was effective against *Saccharomyces cerevisiae*, *Aspergillus niger*, and *Candida* species at the concentration of 1.3 to 1.5 mg/mL [38,41,88,93].

Except for the antibacterial and antifungal effects, chitosan and COS also display antiviral ability. For example, chitosan conjugated with thioglycolic acid can be applied as the carrier for the anti-HIV drug tenofovir to promote the body’s absorption of this drug [94]. Moreover, the sulfated COS derivative can combine tightly with the envelope glycoprotein GP120 of HIV-1 to form a complex, which interrupts the interaction between the GP120 and CD4 cell surface receptor and prevents HIV-1 from entering immune cells [95].

Although the antimicrobial activity of chitosan and COS is lower than traditional antibiotics and antifungal drugs, they are still potential and promising antimicrobial agents which can be applied in in the fields of food, medicine, and agriculture due to their valuable biosafety and biocompatibility.

## 5. The Modulation of the Gut Microbiota by Chitosan and COS

There are trillions of microorganisms in the human gut, most of which are bacteria. They were demonstrated to be closely related to human health [96]. Among the gut microbiota, probiotics refer to the specific microorganisms that can colonize the human body and are beneficial to the host, including *Lactobacillus*, *Bifidobacterium*, *Roseburia* spp., *Faecalibacterium* spp., and *Akkermansia* spp. They promote gut health by regulating the balance of gut microbiota, which improves the functions of the host’s mucosa and immune system [97]. Prebiotics are the organic substances which are not directly digested and absorbed by the host but can selectively increase the abundance and activities of probiotics and be metabolized into beneficial products, mainly short-chain fatty acids (SCFAs), so as to suppress the growth of pathogens and promote the health of the host, mainly including non-starch polysaccharides, resistant starches, and resistant oligosaccharides [98]. Chitin derivatives, containing a large number of β-1,4-glycosidic bonds that can resist digestion by the human gut, share similar characteristics with known prebiotics such as galacto-oligosaccharide, inulin, and β-glucan. This stimulated the interest of researchers in the study of whether chitin and its derivatives have prebiotic potential. In 2002, Lee et al. tested the prebiotic activity of COS in vitro. The findings exhibited that a mixture of COSs with DPs of 2 to 8 significantly promoted the growth of *Bifidobacterium bifidium* and *Lactobacillus* spp. in a dose-dependent manner [99]. In recent years, the prebiotic activity of chitosan and COS has been widely studied and approved, showing an agreeable application potential.

Many animal experiments revealed that chitin derivatives modulate the gut microbiota positively. For example, a study in weaned piglets showed that dietary supplementation with 500 mg/kg/day of COS for 2 weeks improved the composition of ileal and colonic microbiota. Here, the administration of COS increased the amounts of *Bifidobacterium* spp., *Bifidobacterium breve*, *Faecalibacterium prausnitzii*, and *Lactobacillus* spp. in the ileum and colon; *Prevotella* in the ileum; as well as *Fusobacterium prausnitzii*, *Methanobrevibacter smithii*, and *Roseburia* in the colon. However, this significantly decreased the amounts of *Firmicutes* and *Streptococcus* in the ileum and colon; *Bacteroides fragilis*, *Clostridium* spp., *Eubacterium rectale*, and *Methanobrevibacter smithii* in the ileum; as well as *E. coli* in the colon. Meanwhile, COS increased the concentrations of beneficial SCFAs and reduced the concentrations of harmful ammonia and BCFAs in the intestinal luminal content [100]. In another weaned piglets model, the effects of chitosan-chelated zinc on the ileal microbiota challenged with *E. coli* K88 was investigated. The results showed that the piglets treated with a 100 mg zinc and 766 mg chitosan/kg basal diet had significantly increased ileal villus height and ratio of villi height to crypt depth. The administration of chitosan-chelated zinc increased the abundance of *Lactobacillus* and significantly decreased the abundance of *Streptococcus*, *Escherichia-shigella*, *Actinobacillus*, and *Clostridium sensu stricto* 6 in the ileal digesta, indicating the ability of chitosan to modulate the composition of gut microbiota and maintain the function of the intestine [101]. In C57/BL6 mice with azoxymethane and dextran sulfate sodium (AOM/DSS)-induced colorectal cancer (CRC), intragastrical administration of 300 mg/kg/day COS exhibited antitumor activity via reversing the imbalance of bacteria and fungi, especially by reducing the abundance of *Escherichia-Shigella*, *Enterococcus*, and *Turicibacter*, as well as increasing the levels of *Akkermansia*, *Cladosporium*, and butyrate-producing bacteria [102]. Likewise, in a C57BL/6 mice model with DSS-induced colitis, oral administration of COS at 300 mg/kg/day for 7 days increased the abundance of *norank_f_Muribaculaceae*, *Lactobacillus*, and *Alistipes* while decreasing the abundance of *Turicibacte* and the Firmicutes/Bacteroidetes ratio. Furthermore, the levels of propionate and butyrate in the gut were also increased [69]. Another study carried out in HFD-fed male C57BL/6J mice demonstrated that oral administration of COS with a DP of 3 to 5 at 400 mg/kg/day alleviated low-grade inflammation, maintained the intestinal epithelial barrier, and regulated the dysfunctional gut microbiota by significantly decreasing the relative abundance of inflammatory bacteria such as *Erysipelatoclostridium* and *Alistipes*, but increasing the beneficial gut bacteria such as *Akkermansia* and *Gammaproteobacteria* [103]. Additionally, Rahman et al. reported that chitosan protects immunosuppressed C57BL/6 mice from *Cryptosporidium parvum* infection via TLR4/STAT1 signaling pathways and gut microbiota modulation. Oral administration of 1 mg/kg/day chitosan increased the relative abundance of *Bacteroides*, while the abundance of *Tenericutes*, *Defferribacteres*, *Firmicutes*, and endotoxin-bearing *Proteobacteria* decreased [104]. The above studies in C57/BL6 mice significantly support the theory that the anti-inflammatory and immunological activities of chitosan and COS against IBD, CRC, and parasitic diseases depend to a certain extent on regulating the gut microbiota. Tang et al. from our team confirmed that, compared with the HFD containing 5% cellulose (*w*/*w*), the HFD containing 5% chitosan increased the serum leptin level and reduced chronic inflammation and weight gains in C57/BL6 mice. The positive physiological outcomes were further confirmed to be related to the improvement of the gut microbiota by chitosan supplementation via increasing the amount of antiobesity-related species, such as *Coprobacillus cateniformis* and *Clostridium leptum*, and significantly decreasing *Clostridium lactatifermentans* and *Clostridium cocleatum* [105]. We also examined the physiological functions and prebiotic effects of COS in obese C57/BL6 mice induced by HFD. The findings revealed that the diet containing 5% COS (*w*/*w*) markedly inhibited the accumulation of body weight and liver fat induced by HFD and restored the elevated concentration of blood glucose and fasting insulin to normal levels. Next, the analysis of the gut microbiota exhibited that COS specifically enriched *Clostridium paraputrificum*, *Clostridium ramosum*, and *Akkermansia muciniphila* while reducing the abundance of *Clostridium cocleatum* [106]. Moreover, Wang et al. analyzed the antidiabetic effects of COS in mice with type 2 diabetes mellitus (T2DM). They confirmed that intragastrical administration of 140 mg/kg/day COS for 5 weeks can reduce hyperglycemia and hyperlipidemia, prevent obesity, and enhance histological changes in the livers of mice. Meanwhile, this treatment with COS can improve the composition of the gut microbiota by increasing the Firmicutes/Bacteroidetes ratio and the relative abundance of *Verrucomicrobiales*, as well as by decreasing the abundance of *Proteobacteria* [107]. Based on the beneficial effects of the enriched species on weight loss and blood glucose control, these findings suggested that the effects of chitosan and COS on the improvement of metabolic syndromes have a close relation with the modulation of their gut microbiota.

Simultaneously, the in vitro experiments simulating the human gut microbiota also suggest prebiotic effects of chitin derivatives. A study in simulator of the human intestinal microbial ecosystem analyzed the physiological effects of the fungal-cell-wall-derived chitin–glucan complex on the gut microbiota. The results showed that administration of chitin–glucan at 1.5 or 4.5 g/day for 2 weeks produced prebiotic functions by decreasing the Firmicutes/Bacteroidete ratio and increasing the concentrations of *Roseburia* spp. and SCFAs, mainly propionate and butyrate [108]. Via in vitro batch culture fermentation, Liu et al. revealed that COS at the concentration of 30 mg/mL modulated the gut microbiota composition at the phylum level by increasing Bacteroidetes, as well as by decreasing Proteobacteria, Actinobacteria, and the Firmicutes/Bacteroidetes ratio. Further analysis found that COS promoted the generation of beneficial *Bacteroides* and *Faecalibacterium* genera while suppressing the pathogenic *Klebsiella* genus, suggesting an excellent prebiotic effect [109]. Additionally, Wang et al. utilized an in vitro human fecal fermentation mode to analyze the effects of 11 dietary fibers on human fecal microbiota. The results revealed that fecal microbiota regulated by COS was similar to those of fructo-oligosaccharide and inulin, showing positive regulatory effects on beneficial bacteria, including *Parabacteroides distasonis* and *Bifidobacterium* spp. [110]. The studies using in vitro anaerobic batch fermentation models also revealed the relationship between the structure of COS and its prebiotic activity. Mateos-Aparicio et al. studied the effects of COSs with different DAs and found that COS with a high DA of 86% decreased the abundances of *Bifidobacterium* spp., *Eubacterium rectale*, *Clostridium coccoides*, *Clostridium Histolyticum*, *Bacteroides*, and *Prevotella*, suggesting a colonic microbiota imbalance. On the contrary, COS with a low DA of 65% sustained the bacterial abundances above and promoted the growth of *Lactobacillus* and *Enterococcus* [111]. Ji et al. confirmed that a mixture of COSs with DPs of 2 to 6 can specifically enrich propionate- and butyrate-producing microbes, increase the relative abundances of *Faecalibacterium*, *Clostridium sensu stricto* 1, *C. sensu stricto* 13, and *Fusicatenibacter*, as well as increase the content of SCFAs. Meanwhile, the abundance of *Escherichia-shigella* was significantly decreased compared to the control group [112]. They further examined the impact of DP on the prebiotic effect of COS and found that as DP increased, COS significantly promoted the growth of *Bacteroides* and inhibited that of *Proteobacteria* [113]. In brief, the results showed that the prebiotic effect of COS decreases with DA but increases with DP.

The prebiotic effects of chitosan and COS on the gut microbiota in this section are summarized in Table 1.

As described in Section 4.3, chitosan and COS have pleasant antibacterial effects on both Gram-positive and Gram-negative bacteria. However, this resistant effect is probably only directed against the harmful bacteria. Contrarily, when confronted with the probiotics, these chitin derivatives selectively exhibited a growth-promoting effect. This amazing phenomenon has been confirmed. For example, Kipkoech et al. prepared cricket-derived chitosan and evaluated the growth of pathogenic *S. typhimurium* in the presence of beneficial *Lactobacillus* spp. in media containing this chitosan. The consequences demonstrated that chitosan significantly increased the population of *Lactobacillus* spp. but decreased that of *S. typhimurium* [114]. The prebiotic function of chitin derivatives in inhibiting intestinal pathogenic bacteria can be largely ascribed to the inability of most pathogens to utilize them. Therefore, chitosan and COS are preferentially digested and metabolized by the gut prebiotics as a carbon and energy source. The rapidly growing probiotics compete with the harmful bacteria for nutrients and produce specific metabolites with antibacterial effects (e.g., SCFAs), which further inhibit the growth of harmful bacteria. In addition, the results from in vivo animal experiments showed that the effective dose of chitosan to exert prebiotic activity was significantly lower than that of COS. The probable reason for this is that the high MV of chitosan makes its rate and degree of absorption by the small intestine lower than that of COS. Therefore, chitosan remains in the intestine for a longer time than COS and can be metabolized by the gut microbiota to a greater extent.

## 6. Conclusions and Prospects

**Figure 3:**
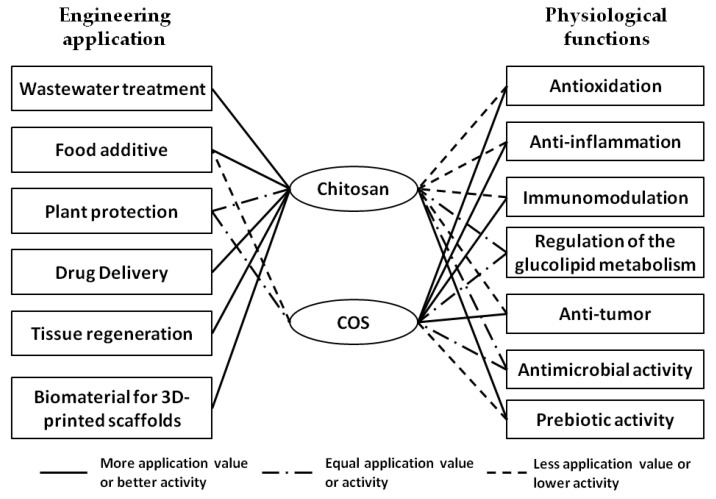
Summary and comparison of engineering applications and physiological functions of chitosan and COS. The straight line indicates that one has more application value or better activity than the other. The dash-dotted line indicates that one has equal application value or activity with the other. The dotted line indicates that one has less application value or lower activity than the other. No line indicates that one has little related application value or activity.

Chitin is one of the most important natural degradable polymers from non-plant sources. Chitosan and COS are the deacetylated derivatives of chitin. In comparison with chitin, chitosan and COS have better solubility and biocompatibility, showing favorable application prospects in industry and agriculture. The safety of chitosan and COS was evaluated by many toxicological tests using in vivo and in vitro models. The results showed that they had no observable short-term toxicity, mutagenicity, or subchronic toxicity [85,115,116,117]. Therefore, chitosan and COS are recognized as Generally Recognized as Safe (GRAS) by the U.S. Food and Drug Administration (U.S. FDA) and are approved as food additives. Other countries, such as the European Union (EU), Australia, New Zealand, and China, have also approved the use of chitosan or COS as food additives or active ingredients in ordinary food and functional food. Chitosan and COS have many biological activities, including antioxidant, anti-inflammatory, and antimicrobial effects, and these activities have been confirmed to be affected by the physicochemical properties (e.g., MV, DD, solubility, and viscosity). Compared with COS, chitosan with high MV and viscosity functions better in drug delivery, wound healing, and tissue repair and regeneration. However, COS shows better anti-inflammatory and immunomodulatory activities due to its stronger solubility and absorbability. Remarkably, the role of chitosan and COS in promoting the proliferation of intestinal beneficial bacteria has attracted much attention, and they are recognized as excellent prebiotics. This is reflected by the number of relevant publications increasing over the years. For example, up to 120 articles from the past five years have been retrieved in PubMed using both chitosan and microbiota as the query terms In contrast to traditional plant-derived prebiotics, chitosan and COS have broader prospects in the prevention and control of noncommunicable diseases, such as cancer and metabolic syndrome, because of their additional biological activities. The engineering applications and physiological functions of chitosan and COS mentioned in this review are summarized in Figure 3.

At present, the biological functions of chitin derivatives are not fully understood, and previous studies on their physiological function were mostly carried out in cell and animal models. Additionally, the studies often used samples containing ingredients with different DPs for analysis, which may create ambiguous or contradictory results. In the future, optimizing the preparation and purification technology of chitosan and COS and improving the knowledge about their biological functions by volunteer experiment and prospective cohort study will provide more clues for the precise treatment of related noncommunicable diseases with chitosan and COS.

## Figures and Tables

**Table 1:** The prebiotic effects of chitosan and COS on the gut microbiota.

Material for Research/Reference	Type of Study	Information about Administration	Major Changes Related to the Gut Microbiota
COS [100]	In vivo trial using weaned piglets	Oral administration at 500 mg/kg/day for 2 weeks	Increase *Bifidobacterium* spp., *Bifidobacterium breve*, *Faecalibacterium prausnitzii*, *Lactobacillus* spp., *Prevotella* in ileum, *Fusobacterium prausnitzii*, *Roseburia* and SCFAs. Decrease *Firmicutes*, *Streptococcus*, *Bacteroides fragilis*, *Clostridium* spp., *Eubacterium rectale*, *E. coli*, ammonia, and BCFAs
Chitosan [101]	In vivo trial using weaned piglets challenged with *E. coli* K88	Treated with 100 mg zinc and 766 mg chitosan/kg basal diet	Increase *Lactobacillus*. Decrease *Streptococcus*, *Escherichia-shigella*, *Actinobacillus*, and *Clostridium sensu stricto* 6
COS [102]	In vivo trial using C57/BL6 mice with CRC	Intragastrical administration at 300 mg/kg/day	Increase *Akkermansia*, *Cladosporium*, and butyrate-producing bacteria. Decrease *Escherichia-Shigella*, *Enterococcus*, and *Turicibacter*
COS [69]	In vivo trial using C57/BL6 mice with colitis	Oral administration at 300 mg/kg/day for 7 days	Increase *norank_f_Muribaculaceae*, *Lactobacillus*, and *Alistipes*. Decrease *Turicibacte* and the Firmicutes/Bacteroidetes ratio
Mixture of COS with the DP of 3 to 5 [103]	In vivo trial using HFD-fed male C57BL/6J mice	Oral administration at 400 mg/kg/day	Increase *Akkermansia* and *Gammaproteobacteria*. Decrease *Erysipelatoclostridium* and *Alistipes*
Chitosan [104]	In vivo trial using immunosuppressed C57BL/6 mice from *C. parvum* infection	Oral administration at 1 mg/kg/day	Increased *Bacteroides*. Decrease *Tenericutes*, *Defferribacteres*, *Firmicutes*, and endotoxin-bearing *Proteobacteria*
Chitosan [105]	In vivo trial using HFD-fed C57BL/6J mice	HFD containing 5% chitosan (*w*/*w*)	Increase *Coprobacillus cateniformis* and *Clostridium leptum*. Decrease *Clostridium lactatifermentans* and *Clostridium cocleatum*
COS [106]	In vivo trial using HFD-induced obese C57BL/6J mice	HFD containing 5% COS (*w*/*w*)	Increase *Clostridium paraputrificum*, *Clostridium ramosum*, and *Akkermansia muciniphila*. Decrease *Clostridium cocleatum*
COS [107]	In vivo trial using mice with T2DM	Intragastrical administration at 140 mg/kg/day for 5 weeks	Increase the Firmicutes/Bacteroidetes ratio and *Verrucomicrobiales*. Decrease *Proteobacteria*
Chitin-glucan complex [108]	In vitro trial simulating the human intestinal microbial ecosystem	Administration at 1.5 or 4.5 g/day for 2 weeks	Increase *Roseburia* spp. and SCFAs. Decrease the Firmicutes/Bacteroidete ratio
COS [109]	In vitro batch culture fermentation with human feces	At a concentration of 30 mg/mL	Increase the Bacteroidetes phylum and the genera of *Bacteroides* and *Faecalibacterium*. Decrease the phyla of Proteobacteria and Actinobacteria, the Firmicutes/Bacteroidetes ratio, the *Klebsiella* genus
COS [110]	In vitro human fecal fermentation mode	At a concentration of 12.5 mg/mL	Increase *Parabacteroides distasonis* and *Bifidobacterium* spp.
COS with the DA of 56% [111]	In vitro human fecal fermentation mode	At a concentration of 10 mg/mL basal media	Sustain *Bifidobacterium* spp., *Eubacterium rectale*, *Clostridium coccoides*, *Clostridium Histolyticum*, *Bacteroides*, and *Prevotella*. Increase *Lactobacillus* and *Enterococcus*
Mixture of COS with the DP of 2 to 6 [112]	In vitro human fecal fermentation mode	At concentrations of 0.4, 2, and 10 mg/mL basal media	Increase the propionate and butyrate-producing microbes, *Faecalibacterium*, *Clostridium sensu stricto* 1, *C. sensu stricto* 13, and *Fusicatenibacter*, as well as the contents of SCFAs. Decrease *Escherichia-shigella*

COS: chitooligosaccharide, CRC: colorectal cancer, DP: degree of polymerization, HFD: high fat diet, T2DM: type 2 diabetes mellitus, DA: degree of acetylation, SCFA: short-chain fatty acid.

## Data Availability

Not applicable.
